# Supplementary material for: Longitudinal monitoring of SARS-CoV-2 spike protein-specific antibody responses in Lower Austria
Source: PLoS One. 2022 Jul 27;17(7):e0271382. doi: 10.1371/journal.pone.0271382 (PMC9328535; doi:10.1371/journal.pone.0271382)
Supplement: Attachment — Submitted filename: RebholzH et al Point-to-Point Reply.docx [file pone.0271382.s002.docx]

**Point-to-point reply to the reviewers**

We would like to thank both reviewers for their very valuable input, which helped us to markedly improve our manuscript!

@Reviewer 1

*“Two important general issues limit the publication of the study and need to be addressed. First, the authors need to better focus the information that this study provides. In fact, it shows the trend of humoral and cellular immunity directed towards a SARS-CoV-2 antigen, namely the spike protein. This information may be of some use in the context of vaccination campaigns with vaccines based on the spike protein. But it cannot be a general biological information on immunity against the virus, because the authors should have evaluated the cellular humoral response against other viral antigens, as carried out in numerous studies (see for example, Le Bert Nature 2020). Therefore, starting with the title and throughout the text, the authors must make it clear that these are humoral and cellular responses against the spike protein and not generically against SARS-CoV-2.”*

We agree with reviewer 1 that our tests addressed the humoral and T cell responses of the SARS-CoV-2 spike protein only, and that the data cannot be easily extrapolated to the SARS-CoV-2 virus. As suggested by reviewer 1 we changed the title of the manuscript, and checked the text of the whole manuscript to be more specific here.

*“Second, the authors should state whether the test they use is calibrated against the WHO International Standard for anti-SARS-CoV-2 immunoglobulin, a standard that the international community has endeavored to produce in order to promote the standardization of SARS-CoV-2 serological methods and allowing for comparison and harmonization of datasets across laboratories. If the test they use is not calibrated against the WHO International Standard for anti-SARS-CoV-2 immunoglobulin, their data is of little use to the scientific community.”*

The serological test used (Euroimmun AG, Lübeck, Germany) was calibrated against the WHO international standard for anti-SARS-CoV-2 immunoglobulin. Tests were performed in a certified diagnostic laboratory (Bioscientia, Ingelheim, Germany), which participates regularly in inter-laboratory performance testing (ring tests), to ensure the highest accuracy and specificity of the applied tests.

@Reviewer 2

*“In this paper Rebholz, Braun et al. sought to assess IgG, IgA and SARS-COV-2-specific INF-gamma release in a cohort of patients and compared these data with same data collected in the same area about 8 months before. Main findings include: i) in patients with previous IgG/IgA positivity, IgG levels reduced at the follow up time, whereas IgA remained stable; ii) no correlation was shown between Ig levels and IFN-gamma response.*

*The authors claim that early T-cell response was associated to mild Covid-19, and yet also discordant data have been reported. The authors should tune their conclusions and discuss their data also in the light of discordant findings (e.g. Tincati et al. Frontiers Immunol 2020).”*

Thanks a lot for this valuable hint! We cited the according references, in order to present a more balanced view on the T cell response related to disease severity.

*“In the methods section, what do the authors mean by “potentially acutely infected but asymptomatic cases”? A more detailed definition of this group of patients is strongly needed.”*

Thanks for the remark, we rephrased the sentence, in order to make it clearer:

The cohort consisted of non-infected participants, and formerly infected but recovered cases. We cannot exclude, that cases were also included with asymptomatic infections at the time point of sample collection.

*“I suggest that the title is changed as in its present form is quite generic and does not really focus on the research presented.”*

We agree with Reviewer 2 that the title is too generic. Indeed, this was also addressed by Reviewer 1. We changed the title accordingly:

Longitudinal monitoring of SARS-CoV-2 spike protein-specific antibody responses in Lower Austria
